# New insights on bridging integrator 1 protein isoforms as a risk increasing gene in Alzheimer’s disease

**DOI:** 10.3389/fnagi.2026.1937726

**Published:** 2026-10-26

**Authors:** Min Hu, Sahar Esmaeeli, Lindsay Stolzenburg, Britney Milkovich, Mariam Jouni, Eugene Nyamugenda, Reza K. Hammond, Peter Reinhardt, Lamiaa Bahnassawy, Laura Gasparini, Jeffrey F. Waring, Aparna Vasanthakumar

**Affiliations:** 1 Genomic Research Center, North Chicago, IL, United States; 2 AbbVie Deutschland GmbH & Co. KG, Neuroscience Discovery, Ludwigshafen, Germany

**Keywords:** Alzheimer’s disease, bridging integrator 1 isoform, clinical trial, gene expression, genome-wide association study by proxy, human induced pluripotent stem cells differentiation, single nucleotide polymorphism

## Abstract

**Introduction:**

Alzheimer’s disease (AD) is a multifactorial, age-associated neurodegenerative disorder that leads to progressive declines in cognition, memory, and the capacity for independent daily living. Genome-wide association studies (GWAS) have identified Bridging Integrator 1 (*BIN1*) as one of the strongest and most reproducible genetic risk factor for AD, which we replicated in our GWAS by proxy (GWAX) analysis on UKBiobank data. However, the mechanism by which *BIN1* impacts AD risk is largely unknown and our work aims to uncover the underlying mechanism.

**Methods:**

We generated human induced pluripotent stem cell (hiPSC)-derived neuronal and microglia cell models to evaluate cell-type specific *BIN1* isoform expression using droplet digital polymerase chain reaction (ddPCR). We then extended our analysis to a Phase 2 AD clinical trial in which we evaluated the association of *BIN1* isoform expression with the *BIN1* genotype status.

**Results:**

Our data indicated that *BIN1* isoform expression patterns shifted with the differentiation of hiPSC into neurons or microglia. We also found that the SNP rs35103166 is associated with the expression of the microglia-specific *BIN1* isoforms.

**Conclusion:**

We demonstrate that *BIN1* genotype is associated with isoform expression, which differs between neurons and microglia, enabling evaluation of disease-specific isoform effects. Given the strong association of *BIN1* with susceptibility to AD, exploring the mechanisms of *BIN1* genetic variants and their impacts on cell-type specific expression could serve as a valuable resource for novel drug discovery.

## 1 Introduction

Alzheimer’s disease (AD) is a complex neurodegenerative disorder associated with age characterized by progressive cognitive decline, memory loss, and impaired daily functioning (Alzheimer et al., 1995; Selkoe and Hardy, 2016). Pathologically, intracellular neurofibrillary tangles of hyperphosphorylated tau and extracellular β -amyloid plaque deposition are two hallmarks in AD brain (Glenner and Wong, 1984; Kosik et al., 1986; Grothe et al., 2017; Kuhlmann et al., 2017; Weller and Budson, 2018). AD is the most common cause of dementia and accounts for up to 80% of all dementia diagnoses (Crous-Bou et al., 2017; Scheltens et al., 2021), affecting millions of individuals worldwide and increasing healthcare cost dramatically (Schaller et al., 2015; Mattap et al., 2022; Tay et al., 2024). Despite extensive research efforts, there is significant unmet medical need for AD treatment. Therefore, there is an urgent need to gain a deeper understanding of the molecular mechanisms involved in AD pathogenesis and disease progression to identify novel therapeutic targets for treating AD.

Studies show that between 60 and 80% of the risk of developing AD is driven by heritable factors (Gatz et al., 2006; Scheltens et al., 2021). Several large genome-wide association studies (GWAS) have identified more than 80 AD-associated genetic risk loci (Lambert et al., 2013; Jansen et al., 2019; Kunkle et al., 2019; Bellenguez et al., 2022; Shade et al., 2024). The Bridging Integrator 1 Protein (*BIN1*) was found to be one of the most robustly replicated genetic susceptibility loci for AD identified through large-scale GWAS and meta-analyses (Seshadri et al., 2010; Hu et al., 2011; Naj et al., 2011; Wijsman et al., 2011; Chapuis et al., 2013; Lambert et al., 2013; Kunkle et al., 2019; Bellenguez et al., 2022).

The BIN1 gene is located on chromosome 2q14.3. It has 20 exons and 14 isoforms derived from alternative splicing of its pre-mRNA, and 11 of these 14 RNA transcripts translate into proteins (Ren et al., 2006; Karch and Goate, 2015; Taga et al., 2020). These isoforms exhibit distinct cellular localizations (Ren et al., 2006; Prendergast et al., 2009; Zhou et al., 2014; Lambert et al., 2022). Although not all research findings indicate consistent isoform distribution patterns, literature indicates that BIN1 isoforms 1, 2, 3, 5 and 7 are expressed in brain, primarily in neurons and astrocytes, referred to as neuronal-astrocytic-specific BIN1 isoforms (Butler et al., 1997; Zhou et al., 2014; Taga et al., 2020). BIN1 isoforms 6, 9, 10 and 12 are predominantly found in microglia, so they are referred to as microglia-specific BIN1 isoforms (Taga et al., 2020; Sudwarts et al., 2022). BIN1 isoforms 4 and 8 are not expressed in the central nervous system (Prokic et al., 2020; Taga et al., 2020), and isoform 8 is mainly expressed in the muscle (Butler et al., 1997; Prokic et al., 2020). This cellular distribution pattern suggests that BIN1 isoforms may have distinct functions in specific cell types and specific brain regions, contributing to the complexity of AD pathogenesis (Chapuis et al., 2013; Sottejeau et al., 2015; Marques-Coelho et al., 2021).

Several studies demonstrate that *BIN1* contributes to AD pathology by either mediating the production and intracellular aggregation of Aβ42 (Guimas Almeida et al., 2018; Zhang et al., 2018; Hu et al., 2021; Bhattacharyya et al., 2022; Jansen et al., 2022; Alsaqati et al., 2023) or by modulating tau pathology (Chapuis et al., 2013; Glennon et al., 2013; Holler et al., 2014; De Rossi et al., 2017; Crotti et al., 2019; McAvoy et al., 2019; Taga et al., 2020). Some studies observed a decrease of *BIN1* isoform 1 and an increase of isoform 9 expression in human AD post-mortem brain samples (Glennon et al., 2013; Holler et al., 2014).

Single nucleotide polymorphisms (SNPs) in BIN1 gene also have been studied for their association with AD risk. Multiple BIN1 SNPs including rs59335482 (Chapuis et al., 2013), rs74490912 (Taga et al., 2020), rs7561528 (Naj et al., 2011; Zhou and Haina, 2017; Bhattacharyya et al., 2022), rs6431223 (Crotti et al., 2019), rs6733839 (Lambert et al., 2013; Crotti et al., 2019), rs754834233 (Perdigão et al., 2021) and rs138047593 (Perdigão et al., 2021) have been identified and reported to influence AD susceptibility, with rs744373 being the most widely investigated (Liu et al., 2013; Tan et al., 2013; Franzmeier et al., 2019, 2022; Han et al., 2019; Qiu et al., 2022; Schaeverbeke et al., 2022).

To better understand the precise mechanisms by which *BIN1* SNPs may contribute to the expression of *BIN1* isoforms and affect AD risk, we first measured the expression of *BIN1* isoforms in hiPSCs) – derived neurons and microglia. We then characterized how genetic *BIN1* SNPs influence *BIN1* isoform expression in a Phase 2 AD trial (NT02880956) and tested their association with AD clinical endpoints in this trial. With this study, we hope to provide new insights on the mechanism by which *BIN1* SNPs and *BIN1* isoforms may contribute to cell type specific expression associated with AD risk, informing novel therapeutic strategies for AD.

## 2 Materials and methods

### 2.1 Stratified case-control association mapping of AD using family history from UK Biobank (UKBB)

A GWAX study using imputed genotypes on the SAIGE (Scalable and Accurate Implementation of Generalized mixed model) platform was conducted, utilizing the family history of AD from UKBB. We thus defined individuals who are not adopted and reported having at least one parent or sibling with AD as proxy cases, while the proxy super controls included subjects 70 years or older who reported no parental history of AD, depression or Parkinson’s disease. Anyone reporting neurological or behavioral disorders was excluded from the control set. We stratified the cases and controls based on their APOE status and performed separate association studies on the subjects who are carriers of APOE e4. The covariates used for these analysis were APOE status, sex, and age, and variants with minor allele count ≥ 10 were included for downstream analysis. Genomic inflation statistics across MAF bins for all GWASs are included in Supplementary Table 2.

### 2.2 hiPSC-differentiation

We used BIONi010-C-13 derived from fibroblast cells using episomal reprogramming (available through EBiSC: www.ebisc.org). Detailed protocols can be found in Supplementary materials and methods.

#### 2.2.1 hiPSC-derived neurons

hiPSCs were cultured with daily media change and differentiated into cortical neural progenitor cells (NPC) through a series of media changes for 17 days. NPCs were further differentiated into neurons after plating in neuronal maintenance media with special supplemental reagents. Two days after neuronal plating, the cells were treated with 0.15 μg/mL mitomycin C to kill dividing cells. Cells for RNA analysis were collected at various differentiation stages, including the hiPSC stage, NPC stage, and neuron. Neuronal differentiation from the same hiPSC line were performed at the same time as ‘replicates’ 4-6 times.

#### 2.2.2 hiPSC-derived microglia

Generation of microglial-like cells was based upon the method by Haenseler et al., (2017). Embryoid bodies generated by dissociating hiPSCs into single cells were plated according to manufacturer’s suggested protocol for 15–18 weeks to generate pre-microglia. These pre-microglia were pooled, counted, and then plated and differentiated into microglial-like cells with microglia differentiation media. Within one to two weeks, differentiating microglial-like cells begin to exhibit typical morphological features. Microglia-like cells were collected for RNA extraction in Trizol (Thermo Fisher Scientific, MA, USA) on day 14 of differentiation. Microglial differentiation from the same hiPSC line were performed independently at different times to generate up to 9 replicates.

### 2.3 Primer design for BIN1 isoforms

Specific primers and probes for *BIN1* isoforms were designed based on Taga et al.’s publication (Taga et al., 2020) using Primer 3 and synthesized by Integrated DNA Technologies (Coralville, IA, USA). Each primer pair and probe are located at the unique exon-exon boundary for that specific *BIN1* isoform with the exception of primer & probe 10 & 12, which detect both isoform 10 and 12, because there is no unique design that can distinguish between these 2 isoforms. See Supplementary Table 1 for detailed information for each primer & probe.

### 2.4 Droplet digital polymerase chain reaction (ddPCR) assay for measuring expression of *BIN1* isoforms

Total RNA was extracted from hiPSCs, pre-microglia, and microglia using the Direct-zol™ RNA MiniPrep Kits (Zymo Research, CA, USA). RNA extraction for neuronal differentiation cells (hiPSCs, NPC, neurons) was done using the RNeasy Mini Kit (Qiagen, MD, USA). RNA from peripheral blood (PB) in the AD trial was isolated using QIAsymphony PAXgene Blood RNA Kit (Qiagen, MD, USA). cDNAs were synthesized using the iScript cDNA Synthesis Kit (Bio-Rad, CA, USA) using 500 ng RNA.

ddPCR was performed following the QX200 Droplet Digital PCR System (Bio-Rad, CA, USA) protocol (Hindson et al., 2011; Miyaoka et al., 2018; Murthy et al., 2023). In brief, droplets were generated using the QX200 AutoDG Droplet Digital PCR System (Bio-Rad, CA, USA). PCR amplification was then conducted on a C1000 Touch Thermal Cycler (Bio-Rad, CA, USA) under the following conditions: 95 °C for 10 min (enzyme activation), 94 °C for 30 s (denaturation) followed by 59 °C for 1 min (annealing/extension). Denaturation and annealing/extension steps were repeated for 39 cycles followed by incubation at 98 °C for 10 min for enzyme deactivation. Finally, the reaction was held at 4 °C for 30 min then 12 °C until droplets were ready for reading. Droplets were read using the QX200 Droplet Reader (Bio-Rad, CA, USA) after PCR amplification. Positive and negative droplets were defined and counted based on the detectable level of fluorescence, reflecting the presence of the target molecule in the droplet. The absolute concentration of *BIN1* isoform expression level was calculated by counting the positive and negative droplets and the counts were fitted to Poisson distribution for statistical analysis in QuantaSoft software (Bio-Rad, CA, USA).

### 2.5 Statistical analysis

All graphs and data analyses were prepared using GraphPad Prism version 10.3 (GraphPad Software, San Diego, CA, USA). All data were shown as mean ± S.D. For the hiPSC differentiated cell line data, ordinary one-way ANOVA with post-testing using Tukey’s multiple comparisons test was used to evaluate statistical differences in the groups. For the PB data from the AD clinical trial, linear regression analyses were performed using age, sex, race, and *APOE* status as covariates. A linear– mixed effects analysis was used to evaluate the association of BIN1 genotype with longitudinal change in clinical scales. Wherever applicable, *P* value < 0.05 was considered statistically significant.

## 3 Results

### 3.1 *BIN1* variants are significantly associated with AD risk in GWAX analysis from UK Biobank (UKBB)

A GWAX analysis was performed in the UKBB cohort by utilizing the family history of AD. As shown in Figure 1A, after inclusion and exclusion criteria were met, we had 55,806 cases versus 122,538 controls. The GWAX analysis identified the most well-known loci associated with AD, including *APOE*, *BIN1*, *TREM2* etc., (Figure 1B). These findings were validated using independent publicly available GWAS data including two manuscripts: the Jansen et al. (2019) and EADB et al. (2026). This comparison is included in Supplementary Table 3. Upon stratifying the subjects using *APOE* status as a co-variate, we found that across all stratifications (*APOE*4+, *APOE*4−, *APOE*3/3 subjects), the *BIN1* locus was identified as most significantly associated with AD risk (Figure 1C and Table 1).

**FIGURE 1:**
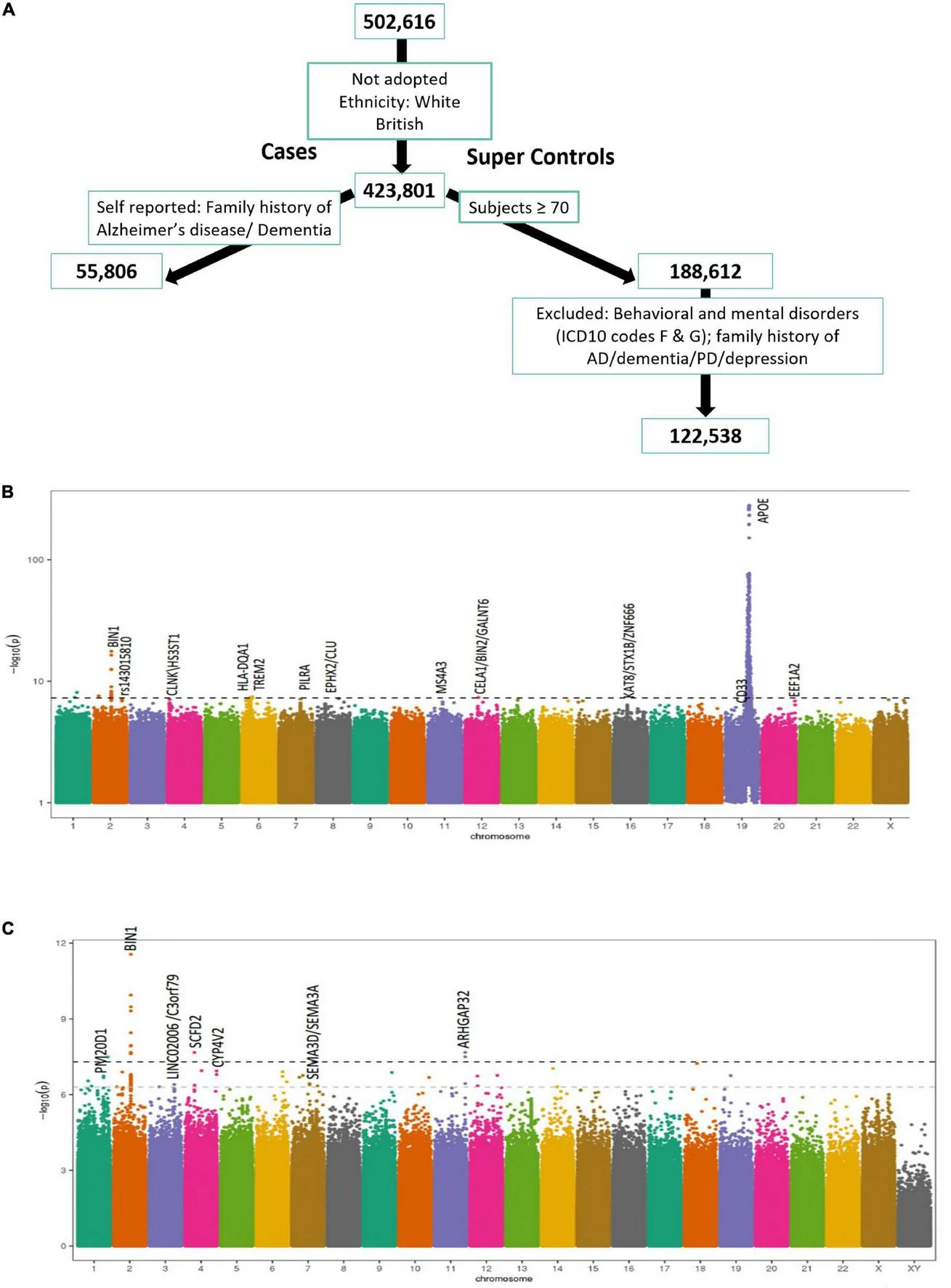
Stratified case-control association mapping of AD using family history from UK Biobank (UKBB). **(A)** A genome-wide association study by proxy (GWAX) using imputed genotypes on SAIGE (Scalable and Accurate Implementation of Generalized mixed model) platform was conducted on 55,806 cases versus 122,538 controls, utilizing the family history of AD from UKBB. Individuals who are not adopted and reported as having at least one parent or sibling with AD were defined as proxy cases, while the proxy super controls include subjects 70 years or older who reported no parental history of AD, depression or Parkinson’s disease. Anyone reporting any neurological or behavioral disorders has been excluded from control set. **(B)** Manhattan plot showing the genome-wide significant hits from the GWAX analysis with chromosome numbers on the X-axis and genome-wide significance on the Y-axis (log_10_(p-value)), showing APOE (chr. 19) as the top significant hit followed by *BIN1* (chr. 2). In addition, data from the cases and controls were stratified based on their *APOE* status, and shown is the association study on carriers of *APOEe4* with Manhattan plot **(C)** with chromosome numbers on the X-axis and genome-wide significance level (-log_10_(p-value)) on the Y-axis shows most significant variants on chr. 2 at the *BIN1* locus. Table 1 showed top significantly associated variants in an *APOE4+* stratified GWAX analysis. SNP: single nucleotide polymorphism MAF: minor allele frequency.

**TABLE 1:** Top significantly associated SNPs in an *APOE4+* stratified GWAX analysis.

AP0E4+					
Unique ID (hg37)	SNP	pval	Beta	MAF	Gene
2:127892810:T:C	rs6733839	2.79E-12	−0.1040716	0.39458055	BINI
11:129214412:A:G	rs560908747	2.11E-08	−3.5967147	0.00010521	ARHGAP32
4:53960000:G:T	rs571695314	2.12E-08	−2.107774	0.00053917	SCFD2
4:188541448:G:T	rs560120139	1.14E-07	−2.4782731	0.00053131	CYP4V2
6:139964185:G:A	rs549317591	1.24E-07	−3.2072627	0.00020876	TXLNB
9:129461828:G:A	rs749654121	1.32E-07	−3.1082817	0.00024491	RALGPS1/LMX1B
1:205878592:GA:G	rs538504793	1.84E-07	−0.1395446	0.07974837	PM20D1
7:84488029:A:C	rs192708784	3.67E-07	−3.680651	0.00015954	SEMA3D/SEMA3A
3:153283580:C:T	rs144523018	3.93E-07	−0.6815993	0.00311122	LINC02006/C3orf79

SNP, single nucleotide polymorphism; APOE e4, apolipoprotein E e4.

### 3.2 Cell-type-specific expression of *BIN1* isoforms in hiPSC models

The mechanism by which *BIN1* SNPs alter AD pathology is still unknown. It is hypothesized that *BIN1* SNPs increase AD risk through their effect on the expression of the *BIN1* isoforms. However, their cell-type specific expression patterns are not as clear. Therefore, we first investigated the distribution pattern of different *BIN1* isoforms in neuronal and microglial cell types using a hiPSC differentiation model.

During hiPSC differentiation into neurons, we observed that the expression of neuronal-astrocytic-specific *BIN1* isoforms (isoforms 1, 2, 3, 5 and 7) increased significantly, with maximal expression at the neuron stage (Figures 2A–E). On the other hand, the expression of microglia-specific *BIN1* isoforms (isoforms 6, 9, 10 and 12) increased significantly in the neuronal progenitor cells (NPC), which then decreased to levels similar to hiPSC when differentiated into neurons (see Figures 2F–H). During the differentiation of hiPSC into microglia, the expression levels of neuronal-astrocytic-specific *BIN1* isoforms decreased during differentiation (Figures 3A–E). Microglia-specific *BIN1* isoforms significantly increased in microglia relative to both the hiPSC and pre-microglia stages (Figures 3F–H). A formal study-power assessment was also performed based on the mean and SD of expression and sample size. All of these tests indicated that for the iPSC models, there was sufficient power to detect differences if present (Supplementary Table 4). Representative images data demonstrated the quality of the cultures obtained in terms of the differentiation of different cell types were shown in Supplementary Figure 1.

**FIGURE 2:**
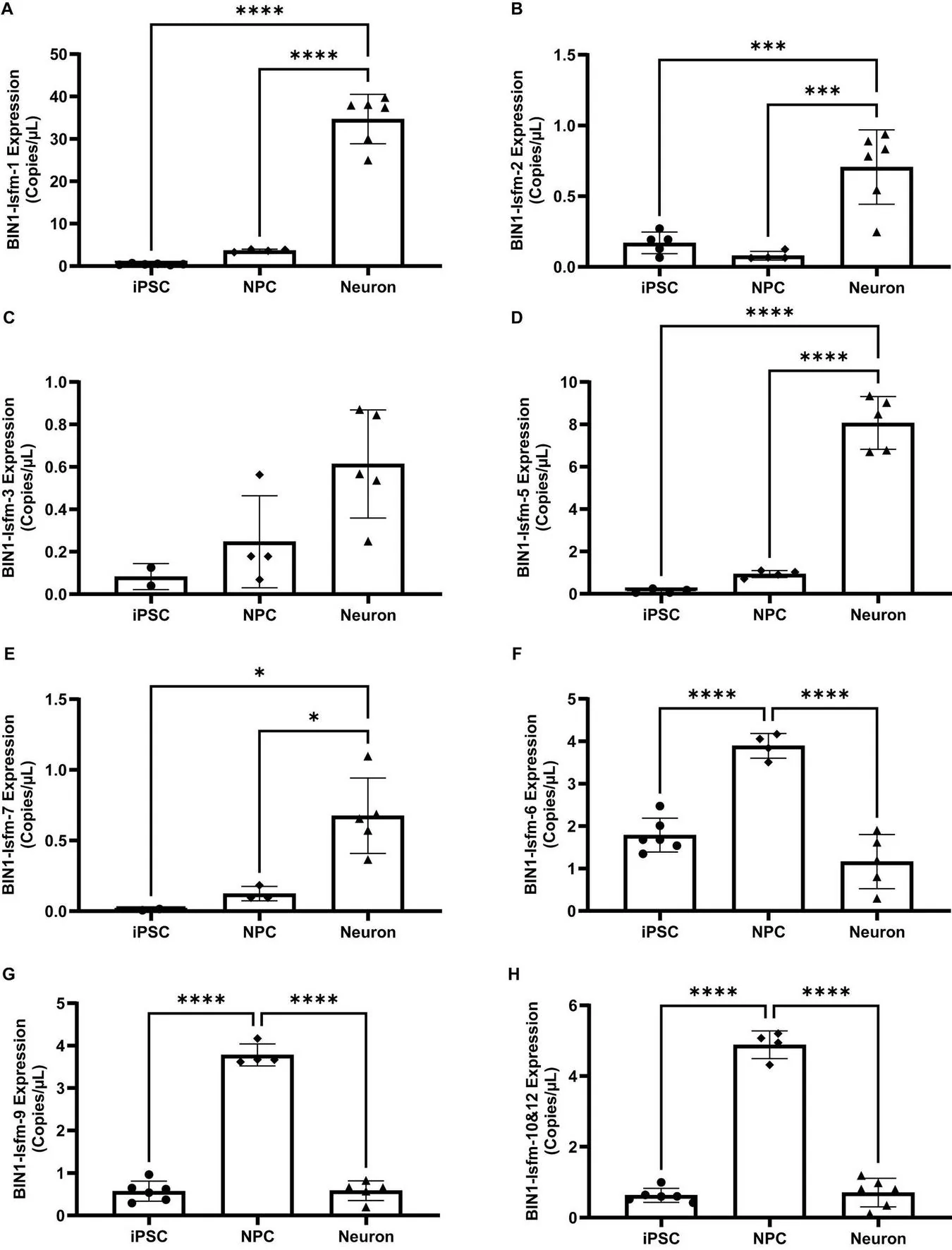
Change in expression level of *BIN1* isoforms during hiPSC differentiation into neurons. hiPSCs were differentiated into neural progenitor cells (NPC), and then into neurons. The expression level of *BIN1* isoforms was measured by ddPCR technology. **(A–E)** Expression level of neuronal-astrocytic-specific *BIN1* isoform 1, 2, 3, 5 and 7, respectively, during differentiation from hiPSCs into NPCs, and finally into neurons. **(F–H)** Expression level of microglia-specific *BIN1* isoform 6, 9 and 10 & 12, respectively, during differentiation from hiPSCs into NPCs and finally into neurons. In each panel, X-axis indicates stage of differentiation, Y-axis indicates *BIN1* isoform expression quantified as copies/μ L. Each point represents the mean ± S.D. of 4–6 independent experiments. Ordinary one-way ANOVA was performed first to compare between groups with ****p < 0.0001. Post-testing was done using Tukey’s multiple comparisons test to compare every group to the other with *p < 0.05; ***p < 0.001;****p < 0.001.

**FIGURE 3:**
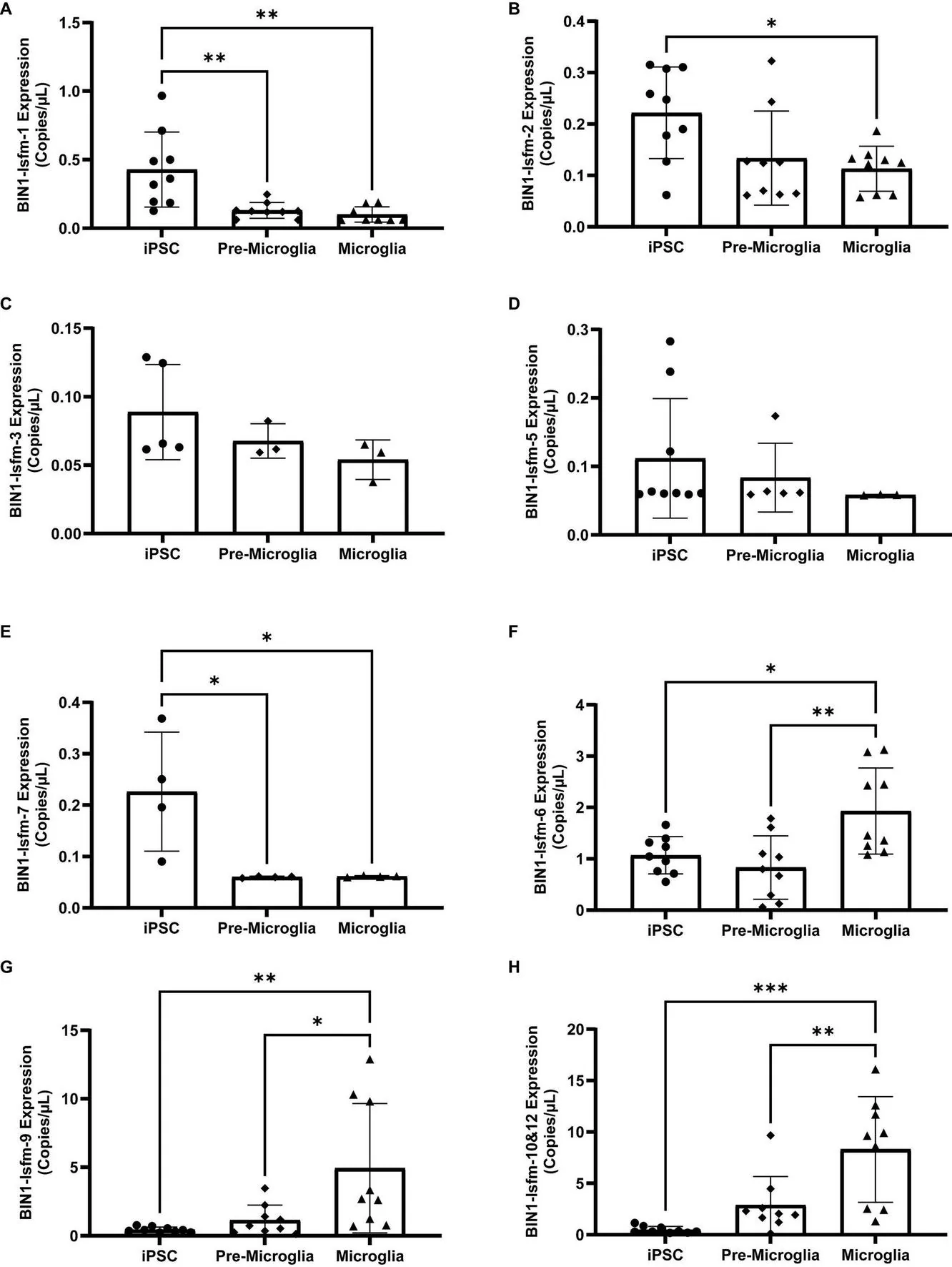
Changes of expression level of *BIN1* isoform during hiPSC differentiated into microglia. hiPSCs were differentiated into pre-microglia first, then differentiation continued to derive microglia. ddPCR technology was applied to detect the expression level of *BIN1* isoforms. **(A–E)** Expression level of neuronal-astrocytic-specific *BIN1* isoform 1, 2, 3, 5 and 7, respectively, during differentiation from hiPSCs into pre-microglia, and finally into microglia. **(F–H)** Expression level of microglia-specific *BIN1* isoform 6, 9 and 10 & 12, respectively, during differentiation from hiPSCs into pre-microglia, and finally into microglia. In each panel, X-axis indicates stage of differentiation, Y-axis indicates *BIN1* isoform expression quantified as copies/μL. Each point represents the mean ± S.D. of 3–9 independent experiments. Ordinary one-way ANOVA was performed first to compare between groups with *p* < 0.0001. Post-testing was done using Tukey’s multiple comparisons test to compare every group to the other with *p < 0.05; **p < 0.01; ***p < 0.001.

### 3.3 Association of the BIN1 SNPs with *BIN1* isoform expression in a phase 2 AD trial

To evaluate whether the expression of *BIN1* isoforms associated with the *BIN1* risk SNPs, we utilized samples collected in a clinical trial for early AD (NCT02880956^1^). This was a trial that tested the efficacy and safety of an anti-tau antibody, Tilavonemab (Florian et al., 2023; Table 2). Genotypes at the *BIN1* loci most significantly associated with risk of AD in our GWAX analyses (rs35103166, rs4663105, rs6733839, rs744373 and rs7561528), all of which are upstream of the *BIN1* gene, were extracted from WGS analyses. We combined both placebo and Tilavonemab-treated patients at Day 1 for the analysis of correlation of these SNPs with the expression of *BIN1* isoforms in PB. Since we are only evaluating cross-sectional association of the expression of *BIN1* isoforms with the SNPs, the treatment arm was not included in the analysis. We only detected microglia-specific *BIN1* isoforms in the PB, given that microglia are brain-specific macrophages. Since our goal was to examine the effect of the alternate allele on downstream expression, we combined the data from the heterozygous and alternate homozygous groups for all our analyses. We performed linear regression analysis to test the association of the SNPs with expression of specific *BIN1* isoforms (isoform 6, 9, 10 & 12) by stratifying patients into alternate allele carriers vs. patients who had the reference homozygous genotype, using age, sex, and *APOE* status as covariates. We found that rs35103166 was significantly associated with increased expression of isoforms 9, and 10 & 12 (Figures 4A–C). A study-power assessment was also performed based on the mean and SD of expression and sample size. All of these tests indicated that there was sufficient power to detect differences if present. (Supplementary Table 4). The other four *BIN1* SNPs (rs4663105, rs6733839, rs744373 and rs7561528) that we tested, including the most studied rs744373, didn’t show any significant association with *BIN1* isoform expression (Supplementary Figure 2).

**TABLE 2:** Demographics of patients in a Phase 2 AD trial (NCT02880956).

	Placebo	Tila-treated
Number	105	315
Age	71.6 +/- 6.7	71.7 +/− 6.5
Sex (F) (%)	62 (59%)	150 (47%)
Race (White) (%)	100 (95%)	304 (96%)
*APOEe4+* (%)	72 (68%)	222 (70%)

Tila, tilavonemab; APOE e4, apolipoprotein E e4.

**FIGURE 4:**
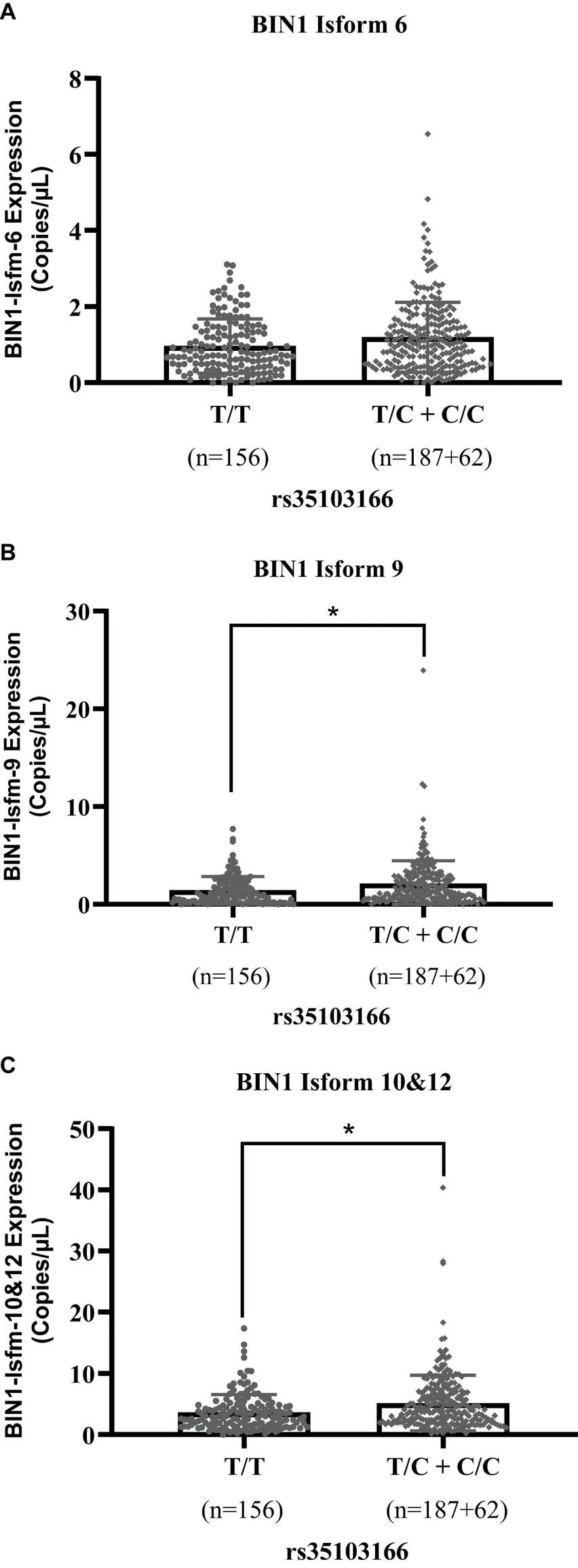
Effect of *BIN1* genotype on expression of *BIN1* isoform in PB from an AD trial. RNA from PB samples collected at baseline from the AD trial (NCT02880956) was used to measure the expression of *BIN1* isoforms using ddPCR technology. The whole genome sequencing data from this trial was used to identify genotype status at *BIN1* SNP rs35103166. At this locus, 156 subjects had reference homozygous (T/T), 187 subjects had heterozygous (T/C) and 62 subjects had alternate homozygous (C/C) genotype. All subjects containing alternate genotypes were grouped together for this analysis. Heterozygous and alternate homozygous subjects were combined into a single alternate-allele carrier group [T/C + C/C, *n* = 249 (187+62)] and compared with reference homozygotes (T/T, *n* = 156). As indicated on the X-axis. Linear regression analysis was performed to test the association of the presence of the SNP with the expression of specific *BIN1* isoforms using age, sex, and *APOE* status as covariates. Each individual panel shows the expression of *BIN1* isoform 6 **(A)**, isoform 9 **(B)**, and isoform 10&12 **(C)**. *p*-values were 0.13, 0.04 and 0.01 for *BIN1* isoform 6, isoform 9, and isoform 10&12, respectively. * indicates *p* < 0.05.

To further understand the impact of the *BIN1* genetic variants on disease, we evaluated the association of *BIN1* rs35103166 with the change in cognitive score using the clinical dementia rating (CDR)-Sum of Box (CDR02-SB) scale, which is commonly used to diagnose dementia due to AD. We specifically evaluated this change in the placebo group so we could identify any changes associated with disease progression over the 2-year timeframe of the clinical trial. When comparing genotype at rs35103166 vs. CDR02-SB at each individual visit, we found no significant associations at baseline or at longitudinal time points (Supplementary Figures 3A–E). To evaluate the longitudinal change in CDR02-SB as it is related to genotype, we performed a linear mixed-effects analysis. This analysis indicated that time since initial visit is highly correlated with CDRO2-SB (*p* = 1.01e-8) while genotype alone is not (*p* = 0.75). However, the interaction term of time by genotype is significantly correlated with CDRO2-SB (*p* = 0.025) (Supplementary Figure 3F and Supplementary Table 5a). These findings suggest a potential genotype-dependent difference in longitudinal cognitive change, although confirmation in larger cohorts will be required. To evaluate whether we had sufficient power to detect differences in our mixed effects model, we performed a formal study-power assessment by running 1000 permutations to calculate the power to detect each variable in the model, which indicated sufficient power for time since initial visit, but that our data were not well-powered to detect differences across genotypes (Supplementary Table 5b).

## 4 Discussion

We have demonstrated that transcript isoforms of *BIN1*, one of the most consistently replicated non-APOE genetic risk loci associated with AD, are distributed in a cell-type specific manner in a hiPSC model differentiated into neurons or microglia. Whereas this reflects previous findings from other models, our work is the first to demonstrate that the expression of these isoforms changes with differentiation of hiPSCs.

Recently, the US Congress put forward the Food and Drug Administration (FDA) Modernization Act 2.0 which recommended the use of alternatives to animal testing, one of which may be hiPSC (Han and Han, 2023). The use of hiPSC has revolutionized our understanding of disease mechanisms and has proved to be a valuable tool for developing novel therapeutics. We were able to effectively demonstrate that differentiation-associated changes in isoform expression can be monitored in an iPSC model system. This provides a foundation for using hiPSC not just to characterize neurodegenerative diseases, but also to monitor expression of specific isoforms during cellular development.

Splice isoforms of *BIN1* have been implicated in the acceleration of AD, potentially through affecting tau aggregation (Zhang et al., 2024), regulating β-secretase cleavage of Aβ precursor protein (APP) and Aβ generation (Bhattacharyya et al., 2022), pro-inflammation (Sudwarts et al., 2022), synaptic dysfunction, and exocytosis of synaptic vesicles (De Rossi et al., 2017; Glennon et al., 2020; Prendecki et al., 2020). Our findings are consistent with the hypothesis that alternative splicing may contribute to the biological effects associated with *BIN1* genetic variation, although direct functional validation remains necessary. Furthermore, we have been able to find differential isoform expression in the PB from AD patients associated with one of the *BIN1* SNPs, suggesting an association of the BIN1 genotype with isoform expression that may relate to disease mechanisms. A schematic illustration of a proposed pathway linking this *BIN1* genetic variant to altered isoform expression and downstream Alzheimer’s disease mechanisms is shown in Supplementary Figure 4.

The impact of variants in the *BIN1* locus has been studied extensively. A recent publication found that microglia specific enhancers harbor a non-coding *BIN1* AD risk variant, the deletion of which led to microglia-specific ablation of *BIN1* expression (Lopes et al., 2022). Loss of *BIN1* has been shown to elevate the production of Aβ42 via BACE1 accumulation at early endosomes (Kaur et al., 2023). The *BIN1* SNP rs744373 has been associated with increased Tau pathology and declining memory performance in the ADNI cohort (Franzmeier et al., 2022). Previous work has also shown that AD brains have an overall decrease in *BIN1* neuronal isoform 1 and an increase in the microglia-specific *BIN1* isoform 9 (Glennon et al., 2013; Holler et al., 2014), which may be due to a shift in brain cell populations, driven by neuronal death and gliosis (Sudwarts et al., 2022). Our work provides support for a hiPSC model to help disentangle the changes are associated with each of the cell types.

Recent publications have shown that dysregulation of mRNA splicing may be a mechanism by which the genetic risk variants play a potential role in disease etiology. Lopes et al demonstrated that known AD risk variants in *CD33* and *MS4A6A* may act through modulation of splicing in the microglia (Lopes et al., 2022). A cross-ancestry atlas of the developing human brain demonstrated that isoform-level regulation mediated a large proportion of GWAS heritability during neurodevelopment (Wen et al., 2024). Exploring this cross-population resource of gene, isoform, and splicing regulation demonstrated that rs35103166, the SNP associated with differential *BIN1* isoform expression in PB, was a splice-quantitative trait locus. Furthermore, this SNP is within a distal enhancer-like signature as identified by the ENCODE project (ENCODE Project Consortium et al., 2020). Lopes et al also demonstrated that candidate causal variants for AD, e.g., USP6NL are within microglia-specific enhancers, and are associated with the expression of the gene in a cell-type specific manner (Lopes et al., 2022). In Meng et al. (2023) this SNP was demonstrated to be highly significantly associated with Hi-C loops, showing high confidence interactions with the *BIN1* gene.

We recognize that there are several limitations to this study. Whereas our findings demonstrate that the BIN1 variant rs35103166 is associated with differential expression of microglia-specific BIN1 isoforms, our study does not establish a causal relationship between altered isoform expression and AD-related cellular phenotypes. The observed associations suggest that modulation of BIN1 isoform expression may represent one of several mechanisms through which genetic variation at the BIN1 locus influences disease susceptibility; however, further functional studies will be required to test this hypothesis directly. In particular, isoform-specific knockdown, overexpression, or genome-editing approaches in relevant cellular models could help determine whether individual BIN1 isoforms influence microglial activation, amyloid-β processing, tau pathology, or inflammatory responses.

Additionally, we used ddPCR, a quantitative readout for the *BIN1* isoforms but it was very limited in the range of detection of splice forms. A long-read method (Heberle et al., 2023), for e.g., nanopore sequencing, would provide an unbiased overview of the various splice isoforms found in the locus.

Another limitation was that only PB samples were available from the clinical trial, and we were therefore only able to detect microglia-specific *BIN1* isoforms, given the low expression of neuronal isoforms in the periphery. Accessing a sample set which includes matched PB and CNS tissue would provide an understanding of the association of *BIN1* SNPs with the expression of the various isoforms. Since we had a smaller number of individual samples analyzed for the clinical trial, we didn’t observe any significant associations of cognitive scores with *BIN1* genotype or isoform expression. A limitation of the present study is the relatively modest sample size available for genotype-stratified analyses, particularly for evaluating associations with longitudinal cognitive outcomes. Formal power analyses indicated that the study had limited power to detect small genotype-associated effects arguing that the absence of significant associations between rs35103166 genotype and longitudinal cognitive decline may not indicate any evidence of no effect. Future studies using larger cohorts and longer follow-up periods will be needed to determine whether BIN1 genotype-associated changes in isoform expression influence clinical disease progression.

In summary, we have been able to demonstrate that a hiPSC-derived neuronal and microglial model can effectively represent biological processes of alternate splicing that may predict disease risk. If the alternate splicing of *BIN1* is the primary mechanism by which *BIN1* risk variants promote disease pathogenesis, this may be a novel target for the development of drugs targeting neurodegenerative diseases (Liu et al., 2022). Since modulation of RNA splicing can be achieved by targeting the components of the spliceosome, or directly targeting the aberrant isoforms (Le et al., 2015), future work will focus on identifying the splice forms most associated with disease mechanisms.

## Data Availability

The data underlying this article will be shared by the corresponding author upon reasonable request, subject to institutional and ethical approval. UKBiobank GWAX summary statistics can only be shared with investigators who have access to UKBiobank.

## Footnotes

1
https://clinicaltrials.gov/study/NCT02880956
