# Supplementary material for: New insights on bridging integrator 1 protein isoforms as a risk increasing gene in Alzheimer’s disease
Source: Front Aging Neurosci. 2026 Oct 26;18:1937726. doi: 10.3389/fnagi.2026.1937726 (PMC13746871; doi:10.3389/fnagi.2026.1937726)
Supplement: Supplementary file 1 [file Data_Sheet_1.pdf]

Supplementary Material

New Insights on Bridging Integrator 1 Protein Isoforms as a Risk Increasing Gene in Alzheimer's Disease

Min Hu[1], Sahar Esmaeeli[1, *], Lindsay Stolzenburg[1, #], Mariam Jouni[1], Eugene Nyamugenda[1], Britney Milkovich[1], Reza K Hammond[1], Peter Reinhardt[2], Lamiaa Bahnassawy[2], Laura Gasparini[2], Jeffrey F. Waring[1], Aparna Vasanthakumar[1]

[1]Genomic Research Center, North Chicago, Illinois, USA

[2]AbbVie Deutschland GmbH & Co. KG, Neuroscience Discovery, Knollstrasse, Germany

[*]Neuron23 Inc., South San Francisco, CA, USA

[#] Northwestern University, Evanston, Illinois, USA

Supplementary Table S1 Primer and probe design

Expressed in Cell T	Name	Forward Primer	Reverse Primer	TagMan Probe
Neuron[26,28]	*BIN1* isoform 1	CCAAGGAAGTCAAGCAGGAG	AAAGTCCAGGTCCAGCAGAC	AGGACACGTTTGTCCCTGAG
Neuron[26,28], Astrocyte[28]	*BIN1* isoform 2	AGAGTCAACCACGAGCCAGA	AAAGTCCAGGTCCAGCAGAC	AGTCCCCATCTCAGTTTGAG
Neuron[26, 28]	*BIN1* isoform 3	GAGTCAACCACGAGCCAGAG	CCAGGACACAGCAAAGGTG	TCTCAGCCCACAGAGAGTCC
Neuron[26,28], Astrocyte[28]	*BIN1* isoform 5	CCAAGGAAGTCAAGCAGGAG	CCAGGACACAGCAAAGGTG	AGGACACGTTTGTCCCTGAG
Neuron[26], Astrocyte[28],Microglia[26,43]	*BIN1* isoform 6	AGGACACGTTTGTCCCTGAG	GGAGGCTGCTTCACTTGC	AGCAGAGGCCTCGGAGGT
Neuron[26,28]	*BIN1* isoform 7	GAGTCAACCACGAGCCAGAG	CCAGGACACAGCAAAGGTG	TCTCAGCCCACAGAGAGTCC
Astrocyte[28],Microglia[26,43]	*BIN1* isoform 9	AGAGTCAACCACGAGCCAGA	GGAGGCTGCTTCACTTGC	AAGTCCCCATCTCAGCCAG
Astrocyte[28],Microglia[26,43]	*BIN1* isoform 10 & 12	GAGTCAACCACGAGCCAGAG	ATTCACAGTTGCTGGGAAGG	AGAGCTCTCTTCCTGCTGTC

Supplementary Table S2: Lambda (genomic inflation statistics) across Minor Allele Frequency (MAF) bins for all GWASs.

GWAS	MAF: [0,0.001)	MAF: [0.001,0.01)	MAF: [0.01,0.05),	MAF: [0.05,1)
**APOE3**	1.004<br>n=30166132	0.986<br>n=7039503	0.999<br>n=2921686	1.037<br>n=7207838
**APOE4**	1.019<br>n=22998953	1.004<br>n=7036898	0.994<br>n=2924403	1.027<br>n=7216587
**APOE2**	NA	0.999<br>n=1533925	0.979<br>n=2929935	0.955<br>n=7208903

Supplementary Table S3: Comparison of top genetic variants in our study with two published GWAS datasets (Jansen et al, Nature Genetics 2019, & EADB., EADI., Bonn., et al Nature Genetics, 2026)

Gene name	rsID	Jansen et al, 2019	EADB., EADI., Bonn., et al 2026	GWAX: all APOE status (this study)
APOE	rs75627662	9.51×10^{-296}	$<1 \times 10^{-300}$	1.7×10^{-280}
BIN1	rs4663105<br>rs6733839	5.46×10^{-26}	1.8×10^{-139}	3.9×10^{-17}
TREM2	rs187370608<br>rs75932628	1.45×10^{-16}	3.08×10^{-38}	5.67×10^{-08}
ZCWPW1	rs7384878	2.38×10^{-10}	4.65×10^{-32}	7.4×10^{-08}
HLA-DRB1	rs6931277<br>rs35472547	1.78×10^{-7}	2.6×10^{-23}	2.1×10^{-07}
CLNK	rs6448451<br>rs10939105	1.19×10^{-8}	1.91×10^{-18}	7.1×10^{-07}
CR1	rs679515	8.85×10^{-10}	6.57×10^{-56}	4.5×10^{-06}
MS4A6A	rs1582763<br>rs7232	4.72×10^{-9}	1.03×10^{-52}	7×10^{-06}
CLU/PTK2B	rs1532278	7.45×10^{-9}	1.39×10^{-47}	7.4×10^{-06}
ADAMTS4	rs4575098<br>rs4233366	6.88×10^{-8}	2.11×10^{-10}	7.9×10^{-06}
ABCA7	rs3752241<br>rs12151021	2.87×10^{-8}	8.19×10^{-39}	8.2×10^{-06}
ECHDC3	rs11257238<br>rs7912495	5.84×10^{-5}	1.72×10^{-24}	2.3×10^{-05}
CD33	rs3865444	4.97×10^{-5}	4.59×10^{-13}	8.9×10^{-05}

Supplementary Table S4: Power assessment

	iPSC Cell Models		PB from Clinical Trial
**BIN1 Isoform**	**iPSC-derived Neuron (Figure 2) (%)**	**iPSC-derived Microglia (Figure 3) (%)**	**rs35103166 (Figure 4) (%)**
**Isoform-1**	100	100	Not detected
**Isoform-2**	99.96	100	Not detected
**Isoform-3**	96.8	90	Not detected
**Isoform-5**	100	80	Not detected
**Isoform-6**	100	100	77
**Isoform-7**	100	100	Not detected
**Isoform-9**	100	100	92
**Isoform-10&12**	100	100	96

Supplementary Table S5a: Cognitive measurements based on longitudinal mixed-effects model

Parameters	Estimate	Standard Error	Degrees of Freedom	t-value	p-value
**Intercept**	2.889558	0.215421	102.593151	13.414	< 2e-16 ***
**Time**	0.035686	0.005549	75.973993	6.431	1.01e-08 ***
**Genotype**	0.084039	0.268498	101.746095	0.313	0.7549
**Time:Genotype**	-0.015566	0.006812	74.737716	-2.285	0.0251*

*** indicates $p<0.001$; * indicates $p<0.05$

Supplementary Table S5b: Power assessment

Parameters	95% Confidence Interval
**Time**	100.0% (99.63, 100.0)
**Genotype**	6.30% (4.87, 7.99)
**Time:Genotype**	59.8% (56.69, 62.86)

Supplementary Figure S1

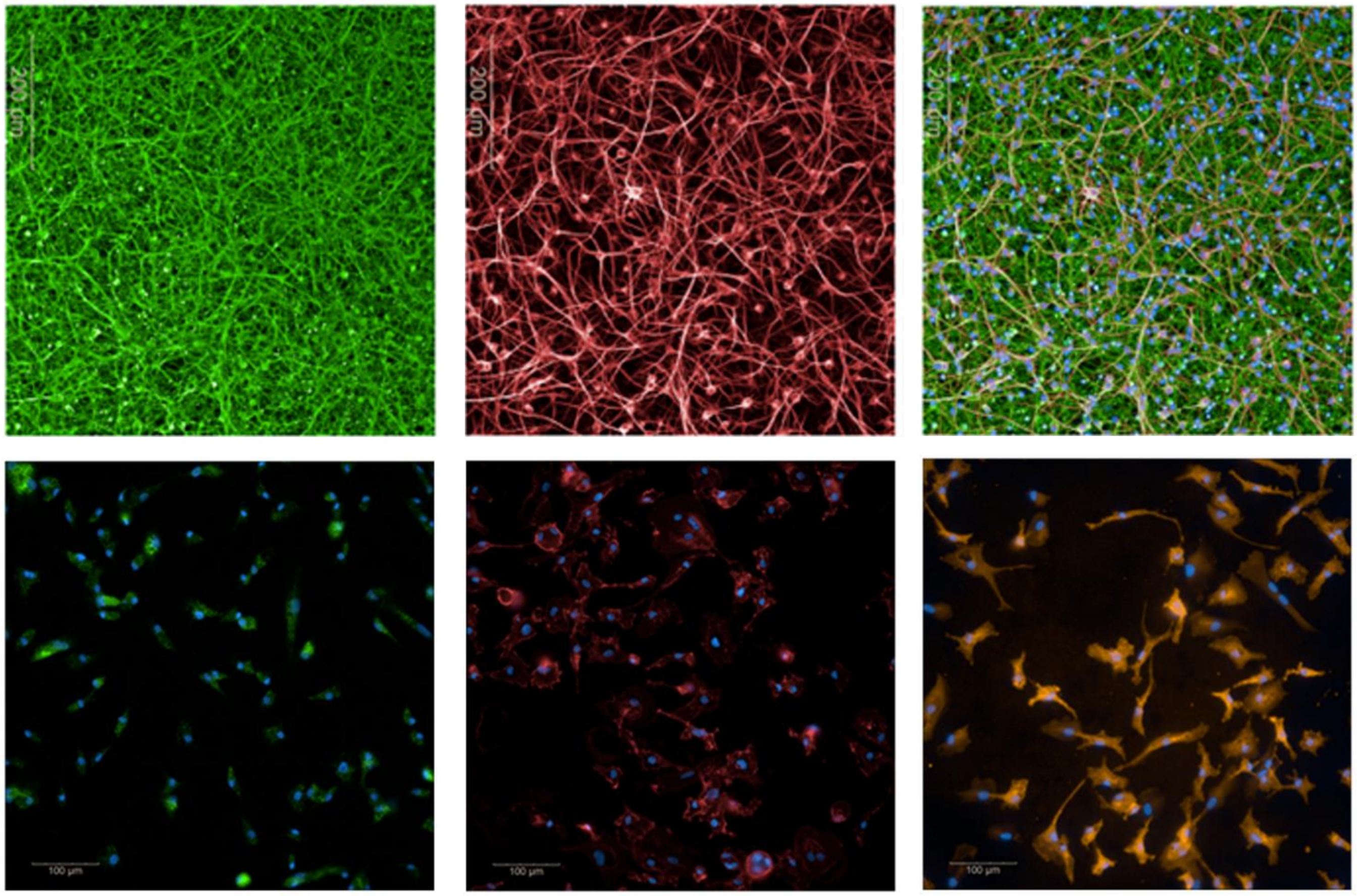

Supplementary Figure S1. Human iPSC-derived neuron and microglia cells.

Top: Representative images of hiPSC- derived neuron at day 21 with staining of β III tubulin (green) and MAP2 (red) for neuron. Merge image shows colocalization of β III tubulin, MAP2 and Hoechst (blue for nuclei) with scale bar 200 µm. **Bottom:** Representative images of hiPSC-derived microglia with colonization of Hoechst staining for nuclei (blue) with TMEM119 (green), CD45 (red) and IBA1 (orange) for microglia with scale bar 100 µm, respectively.

Supplementary Figure S2

(A)

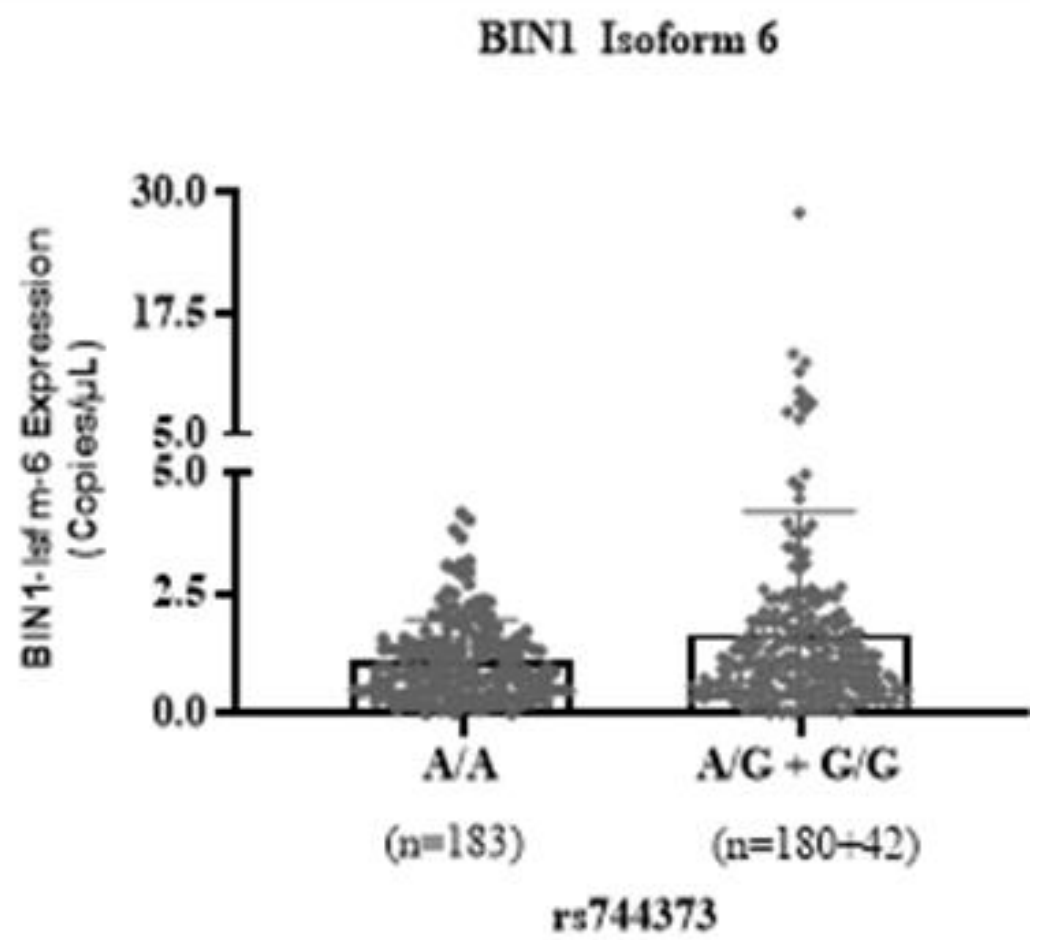

(B)

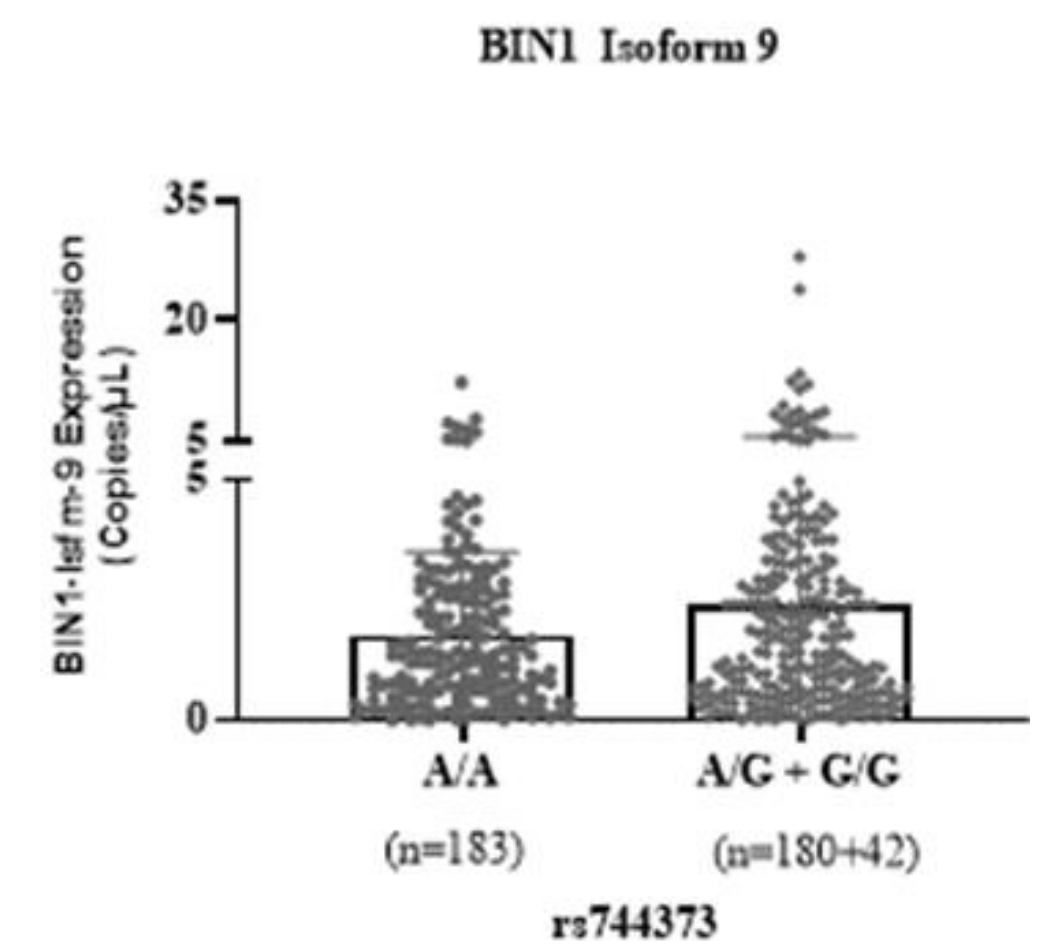

(C)

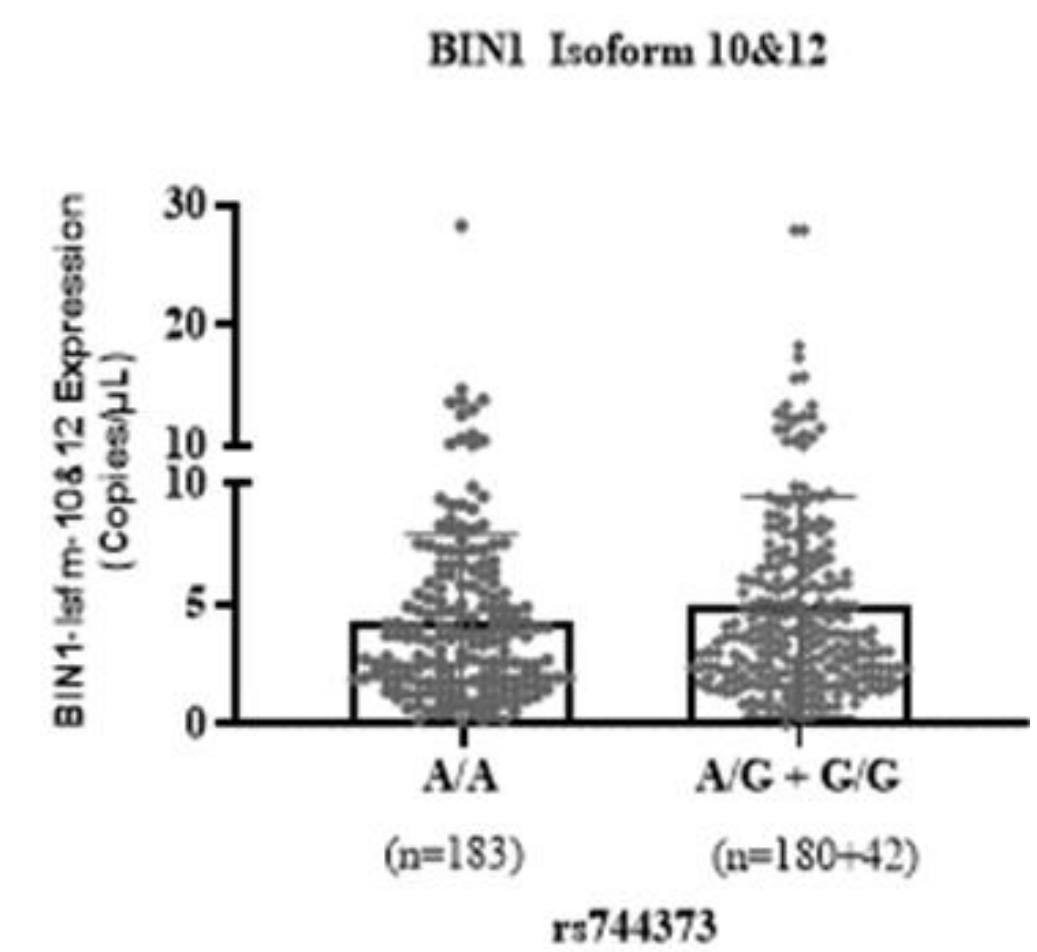

Supplementary Figure S2. Effect of *BIN1* genotype on expression of *BIN1* isoform in peripheral blood from AD trial. RNAs from peripheral blood samples collected at baseline from the AD trial were used to measure the expression of *BIN1* isoforms using ddPCR technology. Whole-genome sequencing data from this trial was used to identify genotype status at *BIN1* SNP rs744373. At this locus, 183 subjects had reference homozygous (A/A), 180 subjects had heterozygous (A/G) and 42 subjects had alternate homozygous (G/G) genotype. All subjects containing alternate genotypes were grouped together for this analysis, as indicated on the X- axis. Y-axis indicates *BIN1* isoform expression quantified as copies/uL. Linear regression analysis was performed to test the association of the presence of the SNP with the expression of specific *BIN1* isoforms using age, sex, race, and *APOE* status as covariates. Each individual panel shows the expression of *BIN1* isoform 6 (A), isoform 9 (B), and isoform 10&12 (C). * indicates $p<0.05$

Supplementary Figure S3

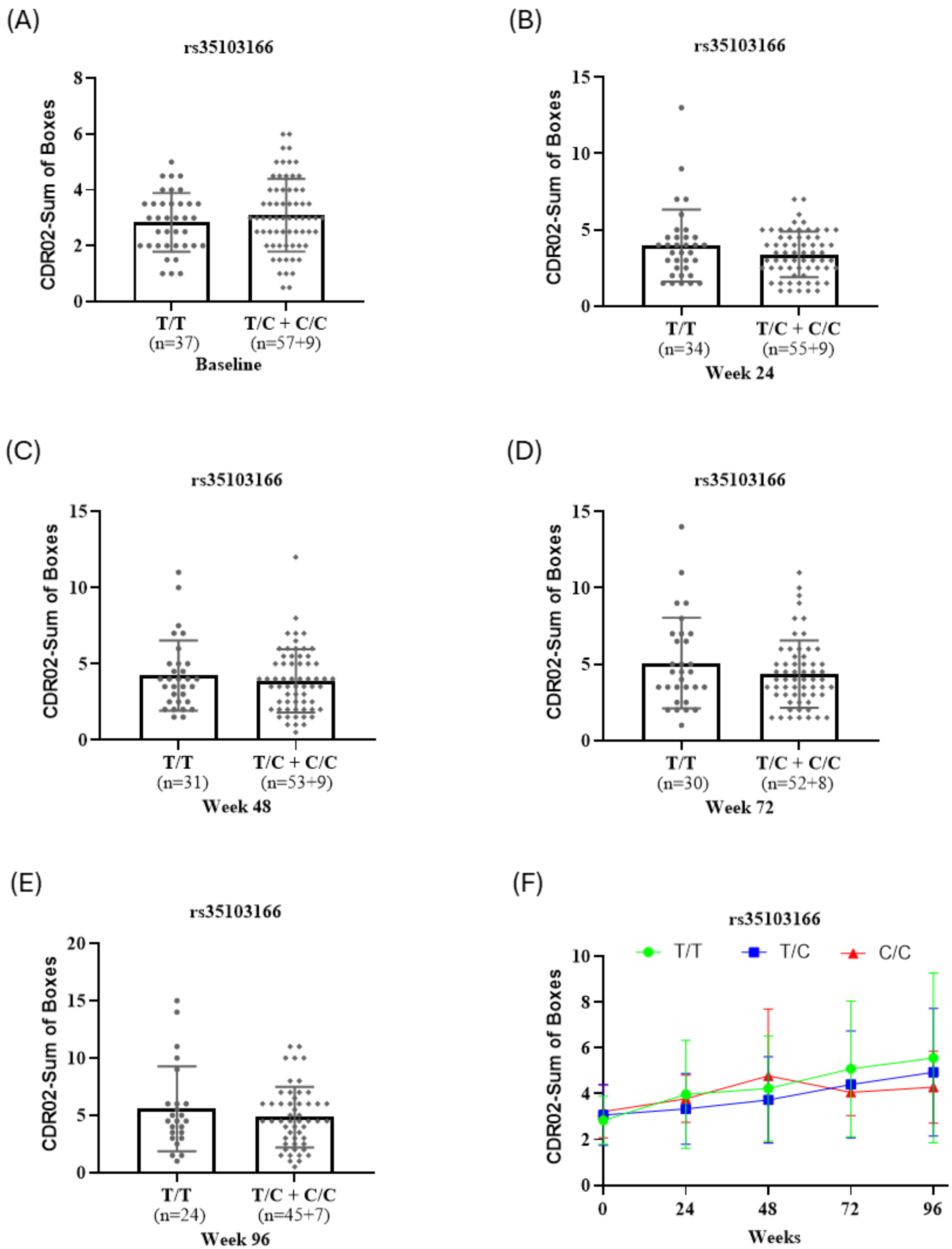

Supplementary Figure S3 Correlation of *BIN1* genotype and AD clinical readout in PB samples from AD trial. PB samples from placebo treated subjects were selected, and ddPCR technology was applied to evaluate the correlation of the *BIN1* SNP rs35103166 with the cognitive test Clinical Dementia Rating (CDR)02- Sum of Boxes. All subjects containing alternate genotypes were grouped together for this analysis. X-axis indicates the different visit time points. Y-axis indicates CDR02-Sum of Boxes. Linear regression analysis was performed to test the association of the presence of the *BIN1* SNP with CDR-sum of boxes at (A) baseline, (B) Week 24, (C) Week 48, (D) Week 72, (E) Week 96 and (F) across all time points using age, sex, race and *APOE* status as covariates.

Supplementary Figure S4 Proposed model linking AD-associated *BIN1* genetic variant to *BIN1* isoform expression and Alzheimer's disease-related biological pathways.

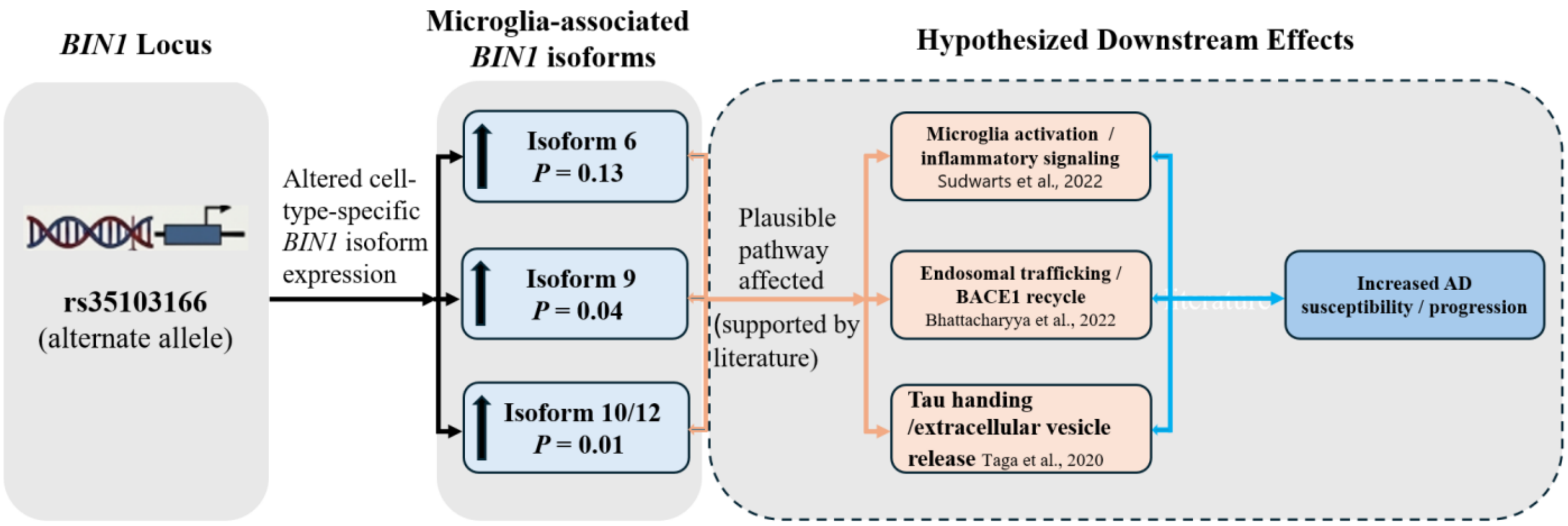

Materials and Methods

Human iPSC-derived neurons

Materials

Product Name	Supplier	Catalog Number
Matrigel® Growth Factor Reduced (GFR) Basement Membrane Matrix, *LDEV-Free	Corning	354230
DMEM/F12, HEPES	Thermo Fisher	11330032
StemFlex™ Complete Medium (basal + supplement)	Thermo Fisher	A3349401
RevitaCell™ Supplement (100X)	Thermo Fisher	A2644501
DPBS, no calcium, no magnesium (CMF-DPBS)	Thermo Fisher	14190136
Accutase	Thermo Fisher	A1110501
Essential 6	Thermo Fisher	A1516401
LDN193189	Stemgent	04-0074
SB431542	Tocris/R&D	1614
InSolution Cyclopamine	Millipore	239806
CHIR99021	R&D	4423
Advanced DMEM/F12	Thermo Fisher	12634010
Neurobasal medium	Thermo Fisher	21103049
Neurobasal Plus medium	Thermo Fisher	A3582901
Pen/Strep	Thermo Fisher	15140122

N-2 Supplement	Thermo Fisher	17502048
B-27 Supplement without vitamin A	Thermo Fisher	12587010
B-27 Plus Supplement (with vitamin A)	Thermo Fisher	A3582801
GlutaMAX	Thermo Fisher	35050061
Y-27632	Millipore	688000
Distilled water (cell culture grade, sterile)	Thermo Fisher	15230147
FGF2	R&D	233-FB-025
DMSO	Sigma	D2650
DPBS	Thermo Fisher	14040133
Laminin	Sigma	L2020
RO4929097	VWR	10192-018
Mitomycin C	Sigma	M4287
BDNF	VWR	10781-158
GDNF	R&D	212-GD-010/CF
Ascorbic acid	Sigma	A4544
Dibutyryl cAMP (dcAMP)	Sigma	D0627

1) Human induced pluripotent cell lines:

In this study we used BIONi010-C-13 derived from fibroblast cells using episomal reprogramming (available through EBiSC: www.ebisc.org).

2) Manual culturing of hiPSCs with daily media change

HiPSCs were cultured in StemFlex complete medium in a T175 flask coated with 77.5 ug/mL Matrigel (Matrigel® Growth Factor Reduced (GFR) Basement Membrane Matrix, *LDEV-Free). The cells were passaged every Monday and Thursday using ReLeSR, at ratios of 1:10-1:20 depending on cell confluency. The cells were cultured in 10 mL/10 cm^2 StemFlex complete medium.

3) Cortical neuron differentiation

1. Generation of cortical neural progenitor cells (NPC)

HiPSC were dissociated as single cells using accutase and seeded at 500,000 cells per cm^2 in 2.5 mL/10 cm^2 of StemFlex complete media supplemented with 10µM Y-27632. The next day (Day0), when the cells were more than 90% confluent, cells were washed twice with DMEM/F12 and the media was replaced with Essential6 media supplemented with 10µM SB431542, 500nM LDN193189, and 500nM CHIR99021 at 5 mL/10cm^2. On Day2, the media was replaced with neural induction media, Essential6 supplemented with 10µM SB431542, 500nM LDN193189, and 5µM Cyclopamine. On day 3, the media was changed to 12ml/10 cm^2 of 0.5x Essential6, 0.5X N2B27-1, 10µM SB431542, 500 nM LDN193189,5µM Cyclopamine, and 1µg/mL Laminin. On Day 6 of differentiation, media was changed to 8ml/10 cm^2 of 0.5x Essential6, 0.5X N2B27-1, 10µM SB431542, 500 nM LDN193189,5µM Cyclopamine, and 1µg/mL Laminin. On day 8, the media was replaced with the same media as day 6. On day 9, cells were washed once with CMF-DPBS and dissociated using 1ml/10cm^2accutase at 37ºC for 15 minutes. The cells were collected in DMEM/F12, centrifuged and resuspended in N2B27-1 +10 µM Y-27632. The cells were counted and replated in a new Matrigel coated dish at 1,500,000 cells per cm^2 in 2.5 mL/10 cm^2 of N2B27-1 +10 µM Y-27632. The following day on Day 10, cells were washed with DMEM/F12, and the media was replaced with N2B27-1(50:50 Advanced DMEM F-12/neurobasal media with 1:200 N2 supplement, 1:100 B27-1 supplement 1% Penicillin/streptomycin and 1x glutamax) supplemented with 10ng/mL FGF2 plus 5µM Cyclopamine. On Day 13 the old media was replaced with N2B27-1 supplemented with 10ng/mL FGF2 plus 5µM Cyclopamine. On day 15, the media was changed to N2B27-1 plus 5µM cyclopamine. On Day 17, neural progenitor cells were frozen in 1 ml N2B27-1 media with 10% DMSO at 10 million cells/vial.

2. Differentiation of NPC into cortical neurons

NPC were thawed and plated in 1 mL of NB27-1 plus 10µM Y-27632 media at 1.33 million cells per well in a 12 well plate (Day-8). The next day the media was changed to NB27-1 supplemented with 500 nM LDN193189 and 5µM Cyclopamine. On Day -5, the media was changed to NB27-1 supplemented with 500 nM LDN193189, 5µM Cyclopamine, and 500 nM RO4929097 (RO). On Day -2, media was changed to neuronal differentiation media (N2B27-1 supplemented with 10ng/mL BDNF, 5ng/mL GDNF, 200 uM ascorbic acid, and 250 uM dcAMP). On Day-2 the plates for plating neurons were coated with 0.5mL of poly-L-ornithine. The next day poly-L-ornithine was aspirated and 1mL of 28 µg/mL Matrigel diluted in cold DMEM/F12 was added. The plates were wrapped in parafilm and stored at 4ºC until the next day for final neuronal plating. Neuronal plating was performed by dissociating the cells with accutase for 20 minutes at 37C. Neurons were plated on poly-L-ornithine + Matrigel plates at 0.5 million cells per well in 1.5 mL of neuronal maintenance media (Neurobasal plus media with 1:50 B27plus, 1% Penicillin/streptomycin, 10 ng/mL BDNF, 5 ng/mL GDNF, 200 µM Ascorbic acid, 250 µM dcAMP and 1 ug/mL Laminin) plus 10 µM Y-27632 and 500 nM RO. Two days after neuronal plating, the cells were treated with 0.15 µg/mL mitomycin C to kill dividing cells. After mitomycin C treatment, ½ media changed was performed on Monday, Friday and Wednesday.

4) Collecting samples for Quantitative Real–time PCR

Cells for RNA analysis were collected in lysis buffer at hiPSC stage, Day 9 replating, Day 17 NPC, DIV0 at neuronal final plating, Day 7 neurons, Day 14 neurons, Day 28, and 6-weeks neurons.

Human iPSC-derived microglia

1) Human induced pluripotent cell lines:

In this study we used BIONi010-C-13 derived from fibroblast cells using episomal reprogramming (available through EBiSC: www.ebisc.org).

2) Culturing of hiPSCs with daily media change

HiPSCs were cultured in mTeSR Plus media (StemCell Technologies) on 6-well plates coated with a 1:600 dilution of Matrigel (hESC-Qualified Matrix, LDEV-free, Corning), and incubated for at least one hour at 37°C. The cells were passaged every 3-4 days using EDTA, at ratios of 1:8, 1:12, or 1:15 depending on cell confluency. To support cell survival, 10µM Y27632 dihydrochloride (ROCK inhibitor, StemCell Technologies) was added to the medium for the first 24 hours following passage. Subsequently, the medium was changed daily without ROCK inhibitor. To maintain all pluripotent or differentiated HiPSC cultures, 2ml of medium per 9.6cm² of surface area or equivalent was used. The passage medium containing the ROCK inhibitor was replaced after 24 hours.

3) Microglia differentiation

Generation of microglial-like cells was based upon the method by Haenseler et al 2017 . Briefly, embryoid bodies were generated by dissociating hiPSCs into single cells utilizing Accutase in a cocktail of 50ng/ml hBMP4 (ThermoFisher), 50ng/ml VEGF (ThermoFisher), and 20ng/ml SCF (Peprotech) with 10µM ROCKi. Cells were then plated at a density of 400,000 cells/well in 2ml mTeSR plus into 24-well Aggrewell 800 (StemCell Technologies) plates according to manufacturer's suggested protocol. Embryoid bodies were then fed every 24 hours with the same media cocktail (without 10µM ROCKi) by applying two 50% media exchanges per well for an additional 4 days.

On the fourth day, the embryoid bodies from each well were filtered to remove any debris and re-suspended in 35ml factory media comprised of Ex-Vivo 15 (Lonza), Glutamax 1:100

(ThermoFisher), Penicillin/Streptomycin 1:100 (Life Technologies), 25ng/ml IL-3 (ThermoFisher), 100ng/ml M-CSF (Peprotech) and 50uM beta-2 mercaptoethanol (BME) (Life Technologies). The cell/cytokine cocktail of each well was then gently transferred to a T175 flask. After two weeks, 20ml of media was removed, and replaced with 20ml fresh factory media. Subsequently each week, 25ml was removed from each factory flask and replaced with 25ml complete factory media. After 5-6 weekly feeds, the embryoid bodies begin to produce the macrophage precursors.

The embryoid bodies produce pre-macrophage precursors for 15-18 weeks. We pooled the pre-macrophage contents of the factory flasks weekly and counted the pre-macrophages, while retaining the embryoid bodies in the flasks. The pool of harvested pre-macrophages was then plated and differentiated into microglial-like cells.

After counting, the pre-macrophages were diluted appropriately with microglia differentiation media comprised of Advanced DMEM/F12 (Life Technologies), N2 supplement 1:100 (Life Technologies), Glutamax 1:100 (ThermoFisher), Penicillin/streptomycin 1:100 (Life Technologies), 50uM 2-mercaptoethanol (2-ME) (Life Technologies), 100ng/ml IL-34 (PeproTech) and 10ng/ml GM-CSF (PeproTech). Cells were plated into 6 well tissue culture plates at 150,000 cells/cm^2. Subsequently a single 50% media exchange was performed every 48-72 hours. Within one to two weeks, differentiating microglial-like cells begin to exhibit typical morphological features. Microglia-like cells were collected for RNA extraction in Trizol (ThermoFisher) on day 14 of differentiation.

Quantitative Real–time PCR

RNA isolation was performed using the Zymo Direct-zol (Zymo) Miniprep RNA extraction kit, following the manufacturer's protocol.
